# The Underlying Cardiovascular Mechanisms of Resuscitation and Injury of REBOA and Partial REBOA 

**DOI:** 10.3389/fphys.2022.871073

**Published:** 2022-05-09

**Authors:** David P. Stonko, Joseph Edwards, Hossam Abdou, Noha N. Elansary, Eric Lang, Samuel G. Savidge, Caitlin W. Hicks, Jonathan J. Morrison

**Affiliations:** ^1^ R. Adams Cowley Shock Trauma Center, University of Maryland Medical System, Baltimore, MD, United States; ^2^ Department of Surgery, Johns Hopkins Hospital, Baltimore, MD, United States; ^3^ Division of Vascular Surgery and Endovascular Therapy, Department of Surgery, Johns Hopkins University School of Medicine, Baltimore, MD, United States

**Keywords:** REBOA, coronary artery flow, vascular trauma, cardiovascular injury, PV loop, partial REBOA

## Abstract

**Introduction:** Resuscitative Endovascular Balloon Occlusion of the Aorta (REBOA) is used for aortic control in hemorrhagic shock despite little quantification of its mechanism of resuscitation or cardiac injury. The goal of this study was to use pressure-volume (PV) loop analysis and direct coronary blood flow measurements to describe the physiologic changes associated with the clinical use of REBOA.

**Methods:** Swine underwent surgical and vascular access to measure left ventricular PV loops and left coronary flow in hemorrhagic shock and subsequent placement of occlusive REBOA, partial REBOA, and no REBOA. PV loop characteristics and coronary flow are compared graphically with PV loops and coronary waveforms, and quantitatively with measures of the end systolic and end pressure volume relationship, and coronary flow parameters, with accounting for multiple comparisons.

**Results:** Hemorrhagic shock was induced in five male swine (mean 53.6 ± 3.6 kg) as demonstrated by reduction of stroke work (baseline: 3.1 vs. shock: 1.2 L*mmHg, *p* < 0.01) and end systolic pressure (ESP; 109.8 vs. 59.6 mmHg, *p* < 0.01). ESP increased with full REBOA (178.4 mmHg; *p* < 0.01), but only moderately with partial REBOA (103.0 mmHg, *p* < 0.01 compared to shock). End systolic elastance was augmented from baseline to shock (1.01 vs. 0.39 ml/mmHg, *p* < 0.01) as well as shock compared to REBOA (4.50 ml/mmHg, *p* < 0.01) and partial REBOA (3.22 ml/mmHg, *p* = 0.01). Percent time in antegrade coronary flow decreased in shock (94%–71.8%, *p* < 0.01) but was rescued with REBOA. Peak flow increased with REBOA (271 vs. shock: 93 ml/min, *p* < 0.01) as did total flow (peak: 2136, baseline: 424 ml/min, *p* < 0.01). REBOA did not augment the end diastolic pressure volume relationship.

**Conclusion:** REBOA increases afterload to facilitate resuscitation, but the penalty is supraphysiologic coronary flows and imposed increase in LV contractility to maintain cardiac output. Partial REBOA balances the increased afterload with improved aortic system compliance to prevent injury.

## Introduction

Non-Compressible Torso Hemorrhage (NCTH) is a significant contributor to early death following trauma, where adjuncts such as tourniquets are ineffective at hemorrhage control (Morrison and Rasmussen, 2012; Morrison et al., 2013a; Kisat et al., 2013; Stannard et al., 2013; Morrison et al., 2014; Morrison, 2017). Historically, emergency department thoracotomy with thoracic aortic cross clamping has been used to temporize patients with NCTH after arrest or in extreme hypovolemic shock (Ivatury et al., 1991; Mazzorana et al., 1994; Burlew et al., 2012; Morrison et al., 2013b; Inaba et al., 2015; Hughes and Perkins, 2020). However, this heroic procedure has poor outcomes and is highly invasive. A minimally invasive endovascular alternative, resuscitative endovascular balloon occlusion of the aorta (REBOA) (Stannard et al., 2011; Morrison et al., 2012; Morrison et al., 2014; Borger van der Burg et al., 2018; Brenner et al., 2018; Glaser et al., 2020; Stokes et al., 2021), which was initially used for management of ruptured aortic aneurysms (Hughes, 1954; Malina et al., 2005; White et al., 2011; Berland et al., 2013), has proved useful for NCTH following trauma. To occlude the aorta, the balloon-bearing catheter is placed through a femoral arterial sheath and inserted into the aorta where it is used to provide endovascular aortic occlusion. This increases afterload and focuses perfusion to the coronary, upper extremity, and head and neck circulation (Madurska et al., 2020; Olsen et al., 2020; Madurska et al., 2021a; Madurska et al., 2021b). The anatomic level of occlusion depends on the clinical scenario and location of hemorrhage (Morrison et al., 2012; Olsen et al., 2020).

Unlike aortic cross clamping, REBOA has an added benefit of being able to be fully occlusive when fully inflated (fREBOA), or only partially filled (pREBOA) and therefore only partially occlusive, thereby allowing some blood flow past the balloon (Dubose, 2017; Sadeghi et al., 2018). Transition between these states is a common though complex clinical situation. Typically, when a patient is in extremis the REBOA will be inserted and made fully occlusive to start (Hughes, 1954; Johnson et al., 2016; Olsen et al., 2020; Russo et al., 2021). In this situation, full REBOA increases afterload thereby permitting hemodynamic rescue, but in turn reduces arterial compliance and can produce supra-physiologic arterial pressures in the perfused organs, while depriving distal organs of flow entirely (Russo et al., 2016; Ribeiro Junior et al., 2018; Wasicek et al., 2019; Nowadly et al., 2020). Maintaining occlusive thoracic REBOA after return of spontaneous circulation (ROSC) or to prevent arrest which is then followed by high arterial pressure therefore places strain on the left ventricle (LV) and is thought to contribute to myocardial injury, and to increase intracranial pressures (Uchino et al., 2016; Johnson et al., 2017; Bailey et al., 2019; Abdou et al., 2021; Edwards et al., 2022). Indeed, clinical and basic science data have shown that fREBOA may be harmful and, therefore, proceduralists will typically transition to pREBOA when feasible (Johnson et al., 2016; Russo et al., 2016; Ribeiro Junior et al., 2018; Sadeghi et al., 2018; Bailey et al., 2019; Wasicek et al., 2019; Abdou et al., 2021). Partial REBOA has been shown to permit blood pressure targeting and produce less circulatory and inflammatory sequelae (Sadeghi et al., 2018) and permits some lower extremity and visceral perfusion, thereby minimizing reperfusion injuries. By allowing some flow beyond the balloon, supra-physiologic arterial pressures are avoided, and reduced strain is transmitted to the LV.

The decision to place a fREBOA in hemorrhagic shock, then transition from fREBOA to pREBOA, and subsequently wean the REBOA to intravenous and vasopressor resuscitation only are well known clinical decisions—here referred to as Critical REBOA Transitions (CRTs). Despite the common clinical application of these CRTs and the abundance of downstream clinical and metabolic data showing pREBOA causes less injury and may be associated with better outcomes, limited data exist to quantify the LV pump biomechanical function or coronary artery flow changes associated with each CRT.

LV pressure-volume (PV) loop analysis and direct coronary artery flow measurements during these CRTs would provide gold standard physiologic data to understand better how REBOA facilitates cardiovascular rescue, but also the underlying mechanisms of subsequent organ injury. The goal of this study is to unravel the cardiophysiologic mechanisms by which REBOA salvages those in hemorrhagic shock and simultaneously causes myocardial injury, and to capture the physiologic evolution between clinically relevant REBOA states. We hypothesize that coronary artery flows and LV PV loop parameters will be associated with REBOA state, with increased derangement with fully occlusive REBOA, compared to partially occlusive REBOA, compared to no REBOA.

## Methods

### Study Overview

Animal studies were approved by the Institutional Animal Care and Use Committee (IACUC) and conform to the National Institutes of Health guidelines for ethical animal research. The study used castrated adolescent male Yorkshire swine (*Sus scrofa*) aged 15–19 weeks old obtained from a local approved USDA vendor (Animal BioTech Industries; Doylestown, PA). The sample size, subspecies choice and sex composition were pre-determined using a power calculation (using G*Power v3.1) (Faul et al., 2009) following a comprehensive literature review performed with the IACUC at our institution using a qualified Librarian. The power analysis was constructed to assess for an effect size of 20% in mean coronary flow and mean ESP, with baseline parameters abstracted from our laboratory experience with swine of this age, weight, and sex (Patel et al., 2021; Stonko et al., 2021; Abdou et al., 2021; Edwards et al., 2021; Stonko et al., 2022). Here, the power analysis suggested the use of at least 4 swine to capture the expected effect in ESP and at least 5 animals for mean coronary flow, so we selected a sample size of 5 to accommodate both outcomes. This literature review also certified that swine are an appropriate animal model to investigate this question. Prior to experimentation, animals were housed in communal pens, under veterinary supervision, with free access to food and water for at least 72 h to allow acclimatization. Animals were fasted for 12 h prior to proceduralization.

**FIGURE 1:**
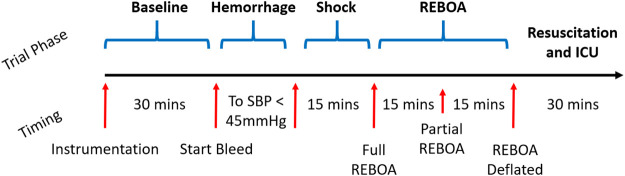
Study Protocol Timeline. After the animals are instrumented and prepared, a 30-min window was observed for baseline data collection. They are then exsanguinated to systolic blood pressure (SBP) of 45 mmHg, and maintained in untreated hemorrhagic shock for 15 total minutes. The REBOA is then inflated and left inflated for 15 min, then partially deflated to 50% aortic occlusion. This is followed by REBOA deflation and then a 30-min ICU period where the animal is further resuscitated and additional labs are acquired.

The study protocol consisted of four overall phases: Instrumentation and baseline data collection phase, exsanguination and shock phase, a REBOA phase divided into full and partial REBOA subphases, and a recovery/resuscitation and ICU phase (Figure 1). A full, step-by-step study protocol was published before scientific work was conducted, and is available on the Protocol Exchange (Uchino et al., 2016). This protocol was conducted as approved by the local IACUC: 0821007.

## Animal Protocol

### Animal Instrumentation and Monitoring

**FIGURE 2:**
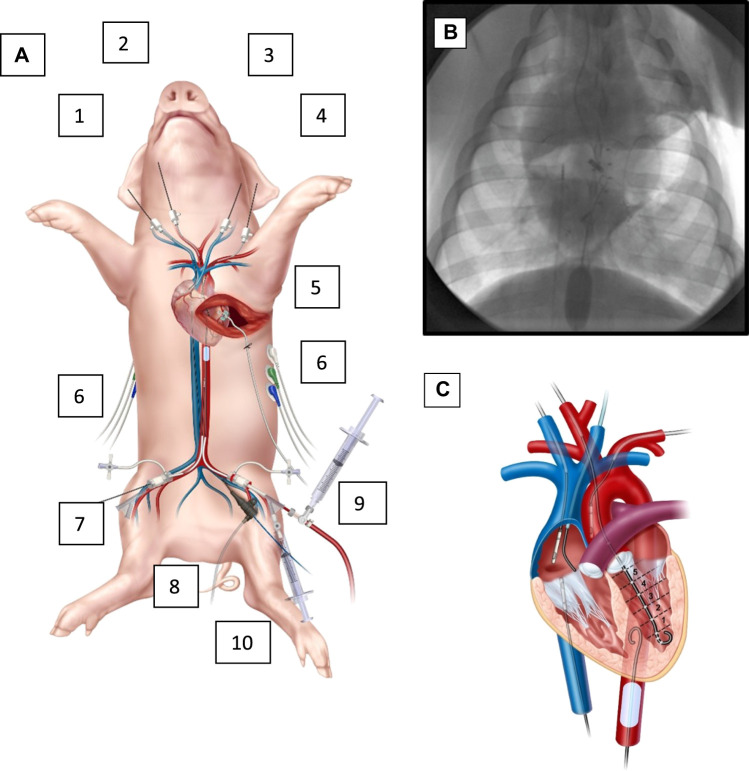
Animal instrumentation and overview. **(A)**. Instrumented animal. (1) right IJ with 7 french (fr.) cannula in right atrium for central venous gases, (2) right carotid with 7 fr. cannula with left ventricle PV catheter, (3) left IJ with right atrial pressure monitor and (4) left brachial artery with aortic (above the REBOA) pressure monitoring. Also, with an antero-lateral thoracotomy with a dissected left coronary; (5) a flow probe around the left coronary, and (6) EKG leads on left and right posterior chest. In the lower extremities, (7) right femoral artery with 7 fr sheath for an aortic pressure (below the REBOA), (8) a right femoral vein central venous catheter for controlled exsanguination, and (9) left femoral artery with 9 fr. sheath for the REBOA catheter and (10) left femoral vein 7 fr. Cannula for transfusions, medications, and IV fluids as needed. The animal also has a rectal temperature probe, endotracheal tube and oxygen saturation probe (not shown). **(B)**. Fluoroscopic image of the chest while instrumented, with the REBOA inflated. From above (left) an RA catheter for central venous gases, and (right) an aortic pressure catheter (above the level of the REBOA), a central coronary flow probe with wire looped extra-corporally. From below, (left) CVP catheter in IVC, and (right) inflated REBOA in zone 1 of descending aorta. Not pictured is the abdominal aortic pressure balloon, which is below the REBOA. **(C)**. Cartoon illustration of the central instrumentation. PV catheter in the LV, IVC pressure catheter, CVP catheter, and aortic pressure monitors, as well as inflated REBOA and RA catheter.

Animals were sedated with Telazol (5 mg/kg)/Xylazine (2 mg/kg) *via* intramuscular injection. General anesthesia was induced and maintained with Isoflurane by facemask followed by orotracheal intubation. The animals were placed on a warming blanket set to 37°C. The animals were mechanically ventilated with a targeted fraction of inspired oxygen of 40% which is monitored continuously with pulse oximetry as well as confirmed at baseline, hourly, and as needed clinically with serial arterial blood gases to examine the PaO_2_ and SaO_2_. Arterial blood gases were also used to maintain an end tidal pCO_2_ of 30–45 mmHg. No animals in this study needed additional respiratory management or has respiratory complications. Instrumentation of the animal involved percutaneous access *via* the Seldinger technique and cannulation of four major arteries for placement of a PV loop catheter through a carotid artery (Transonic Corporation, Ithaca, NY), an ER-REBOA Plus *via* an iliac artery through common femoral access (Prytime Medical, Boerne, TX), and for continuous aortic pressure monitors above and below the REBOA catheter via the other iliac artery (below) and either the other carotid artery or a brachial artery (above); four major veins for controlled hemorrhage, CVP monitoring, central venous gases, and transfusions and medications; and an antero-lateral thoracotomy with left coronary dissection for the surgical placement of a arterial flow probe, Figure 2. The coronary flow probe (Transonic Corporation, Ithaca, NY) was placed through the thoracotomy incision under direct observation: the left coronary artery was dissected carefully off the heart, looped circumferentially with vessel loops and a 3 mm probe was placed circumferentially around the artery. Catheter, pressure monitors and the PV loop catheter location were set and confirmed with C-arm fluoroscopy (OEC 9800, General Electric, Boston, United States). Finally, a urinary cystostomy was performed for urinary drainage. The animals were monitored with electrocardiography (ECG), temperature probes, pulse oximetry, and real time arterial pressure tracings via the aforementioned vascular access sites.

### Baseline Data Acquisition and Exsanguination Periods

Once the animals were instrumented according to the above strategy, they were given 50 mg of dextrose in 1 L of crystalloid and allowed to equilibrate. They were then monitored for a baseline period of 30 min. During this baseline period a venous blood gas, arterial blood gas (Radiometer, Copenhagen, Denmark), troponin (ng/ml) (Abbott Labs, Chicago, IL), and Chem8+, including hematocrit (%) and hemoglobin (mg/dl) (Abbott Labs, Chicago, IL) were obtained. Twenty-five cubic centimeters of whole blood was obtained and centrifuged in red-topped tubes (Becton Dickinson, Franklin Lakes, NJ). Then, serum was extracted and stored in negative 5-degree Celsius freezer. To facilitate a controlled exsanguination, one of the lower extremity venous cannulas was connected to a peristaltic pump. Blood was removed *via* the peristaltic pump at 50 ml/s *via* the venous cannula until reaching a systolic blood pressure goal of 45 mmHg. Following exsanguination to target, a 15-minute period of shock followed where the animal was not resuscitated. At the end of this period repeat venous blood gas and resistivity were collected. If necessary for ventilatory management, an arterial blood gas was collected at this time also.

### Resuscitative Endovascular Balloon Occlusion of the Aorta Period

After the aforementioned exsanguination period and 15-minute period of un-resuscitated shock, an ER-REBOA was placed *via* one of the iliac arteries to full aortic occlusion, confirmed with continuous pressure monitoring of the aorta above and below the balloon and fluoroscopic confirmation, Figure 2. Over the next 15 min, the animals were allowed to equilibrate with the REBOA with full aortic occlusion in place.

Next, the REBOA was partially deflated to maintain a target systolic pressure differential of at least 50%. At this point, resuscitation was started, and the animals were transfused the previously shed blood, and, if necessary, up to 3 L of crystalloid fluid. Resuscitation goals also included maintenance of a systolic blood pressure greater than 65 mmHg, as outlined in detail below. Glucose less than 65 mg/dl was treated with dextrose, and pCO_2_ was addressed with mechanical ventilatory changes as appropriate. The protocol published with the IACUC called for management of a pH of less than 7.2 on any ABG other than the final ABG to be treated with bicarbonate, however no animals required this therapy. In this study, animals were placed into and maintained in hemorrhagic shock for the first two phases of the study. Here, no vasoactive medications are indicated because these are pre-injury (baseline) and pre-hospital (shock) phases. Then, hypotension was treated with an occlusive REBOA and during this phase animals universally became transiently hypertensive and did not require vasopressors. However, as animals entered the pREBOA and resuscitation phases, they were transitioned to transfusion with their shed blood back followed by up to 3 L of IVF, as mentioned above. If during this period the animals had a sustained MAP <65, a pressure infuser bag (Medline, Northfield, IL) was used to deliver fluids more rapidly. Here, no more than the one blood bag plus one 1 L bag of IV fluid was delivered concomitantly, and when the blood was completed, then only one bag of IV fluid was delivered at a time. If this was still unable to sustain a MAP >65, or when the shed blood plus 2 L of IVF was given and MAP was not sustained with transition from resuscitative fluids to maintenance fluids, then Norepinephrine was the pressor of choice for the was used to maintained MAP above 65 mmHg with carrier IVF computed to complete the third liter in the remaining time within the study, Here, Norepinephrine was titrated with a digital syringe pump (digiPump SR31x, Digicare Animal Health, Boynton Beach, Florida) and carried into a femoral access site with a 0.9% Normal Saline carrier as described. In no cases were second line pressors required.

After this 15-minute period of partial REBOA, the REBOA was then fully deflated. Repeat blood was collected and treated as above, and the animal was monitored for at least 30 min with continued resuscitation as needed. Prior to termination at the end of this period repeat labs were obtained once more.

## Data and Statistical Analysis

### Data Capture and Analysis

Recorded animal data included weight, exsanguinated blood volume and baseline blood resistivity (Transonic Corporation, Ithaca, NY). Using aforementioned animal instruments, physiologic data, including, oxygen saturation (SaO_2_), end-tidal carbon dioxide (ETCO_2_), core body temperature, electrocardiography, aortic arch and abdominal aortic pressure, and central venous pressure, heart rate (HR), were recorded continuously for analysis.

Pressure and flow data was acquired and recorded using software (PowerLab and LabChart, ADInsturements, Sydney, Australia). Coronary flows are measured continuously and recorded in microseconds, though extracted in 5 ms increments. The pressure-volume loops are generated with a micromanometer tipped catheter (Transonic Corporation, Ithaca, NY) and computed with the admittance method (Johnson et al., 2017; Edwards et al., 2022). PV loop data was recorded in pressure versus volume versus time data with LabChart, and exported to MATLAB (Mathworks, Nantick, MA, United States).

### PV Loop and Coronary Flow Analysis

LabChart analyzes each PV loop, which corresponds to one cardiac cycle, for hemodynamic parameters such as stroke work, heart rate, and measures of preload, afterload and (Heindl et al., 2020; Edwards et al., 2022) contractility over time. We have previously described methodology for averaging the PV loop over a time period to determine a single PV loop that is representative of LV function during that time period, and published Matlab code to execute this procedure (Edwards et al., 2022). In summary of the mathematical methodology, we convert raw pressure-volume data to polar coordinates, interpolate numerous cardiac cycle loops, then convert back to cartesian coordinates. This provides an average PV loop. From this loop, we can compute the end systolic pressure volume relationship (ESPVR) and end diastolic pressure volume relationship (EDPVR) and from these average hemodynamic measures including cardiac output, end systolic pressure (ESP), end diastolic pressure (EDP), end systolic volume (ESV), end diastolic volume (EDV), as well as ejection fraction (EF), stroke work (SW) and volume (SV) and arterial elastance (Ea).

Coronary flow versus time in milliseconds was also pulled directly from LabChart and exported to Matlab. Here it was plotted as flow (ml/min) versus time (in 5 ms timesteps). These waveforms were then compared by examining their peak heights (peak flow), the percent time spent negative (in retrograde coronary flow), and the net area under the flow curve (total forward flow per cardiac cycle, then scaled across the examined time interval).

Mean systolic pressures and parameters from the PV loop were compared by t-tests comparing parameters from Baseline to Shock, then from Shock to all other study periods pairwise, with one-way repeated measures ANOVA accounting with multiple comparisons with Bonferroni’s correction. Contractility (Ees) is compared from one study period to the next study period across the CRTs, but also for return to Baseline and Shock, and each of these was tested with one-way ANOVA also with multiple comparisons accounted for using Tukey’s honestly significant difference (HSD) test. Coronary flow tracings were examined qualitatively by plotting left coronary flow versus time for each study period, and quantitatively by examining important features of the waveforms: mean peak coronary flow, mean percent time in flow reversal, and mean area under the coronary flow curve. These were compared statistically using paired t-tests between study periods in the order in which they occurred. Across both PV loop parameters and coronary flow metrics, means were reported and plotted with standard deviations as error bars. Lactic acid distributions were non-normal and at different study periods were compared using the Kruskal-Wallis with reporting of medians and interquartile ranges. *p* < 0.05 was considered statistically significant after accounting for multiple comparisons*.* After data extraction, some data was saved in Excel R. 2103 (Microsoft, Redmond, WA), and post-processed for figure creation GraphPad Prism v. 9.2.0 (GraphPad Software, San Diego, CA).

### Histologic Analysis

At the end of the study animals were euthanized as previously described. Terminal blood was collected and immediately following euthanasia full thickness myocardial samples from the left coronary distribution of the left ventricular free wall were obtained from all animals and fixed in 10% formalin solution (Sigma-Aldrich, St. Louis, MO).

Samples were stained with Hematoxylin and Eosin (H&E), which were prepared by designated pathology assistants and examined by a board-certified pathologist, and a report was generated for each sample. Samples were imaged with ×40 magnification, and figure generation was performed with Aperio ImageScope (Leica Biosystems, Deer Park, IL).

## Results

**FIGURE 3:**
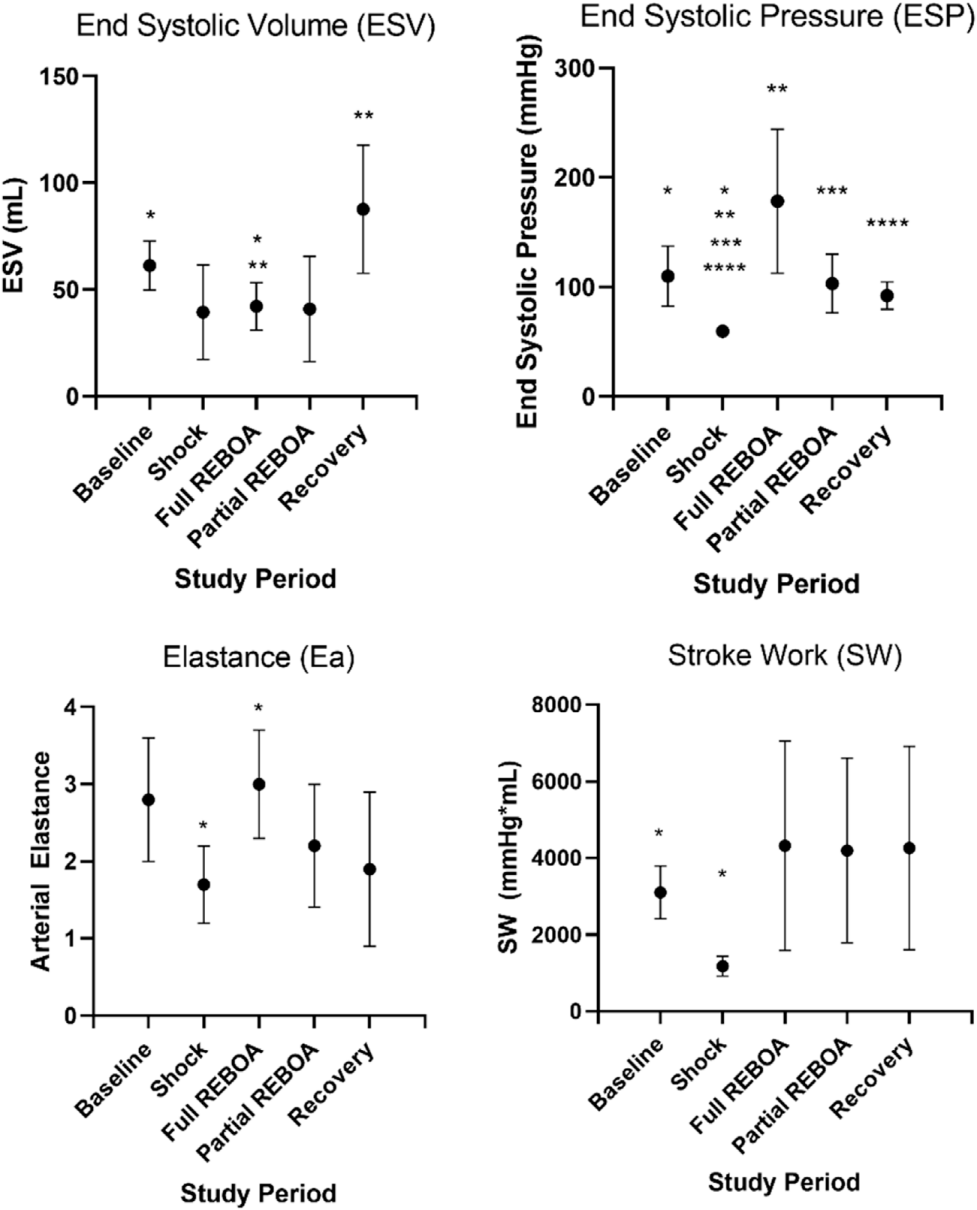
Select left ventricular functional parameters from Supplementary Table S1A. Here SW, Ea, ESV and ESP are plotted against study period. Asterisks denote statistical significance between parameters across study periods, *p* values provided in Supplementary Table S1B.

Five swine (mean 53.6 ± 3.6 kg) underwent the study protocol. All five animals were successfully instrumented fully and bled to a systolic pressure of 45 mmHg without complication. Hemorrhagic shock was induced and maintained as demonstrated with reduction in Baseline to Shock periods of mean SW (3.1 ± 0.69 L*mmHg vs. 1.2 ± 0.26 L*mmHg, *p* < 0.01) and average ESP (109.8 ± 27.5 mmHg vs. 59.6 ± 5.5 mmHg, *p* < 0.01), Figure 3 and Supplementary Table S1. Arterial blood gases were drawn during Baseline, after REBOA placement and at the end of study, Table 1. From Baseline to Post-REBOA, lactate peaked from median 1.5 (1–3.5) mmol/L to median 6.9 (3.5–12.2) mmol/L (*p* = 0.01), Table 1 and Supplementary Figure S2. From Baseline to post-REBOA period pH dropped (7.44 vs. 7.19, *p* = 0.01). There was no further deterioration in pH, change in lactate or other parameter from post-REBOA to the end of the study (all *p* > 0.05), Table 1. Troponin also did not change from Baseline to End of Study (*p* = 0.11). Blood was drawn serially for serum chemistries, and the animals stayed in appropriate clinical ranges throughout the study, Table 2.

**TABLE 1:** Selected study clinical laboratory values. Arterial Blood Gas (ABG) and Troponin from each study period, with comparisons between Baseline and Post-REBOA, and between Post-REBOA and the End of Study.

Selected laboratory values	Baseline		After REBOA		*p*-value	End of study		*p*-value
Arterial blood gas	Mean	SD	Mean	SD	Baseline-REBOA	Mean	SD	REBOA-end
pH	7.44	0.09	7.19	0.08	0.01	7.10	0.06	0.21
pCO_2_ mmHg	37.22	2.11	48.34	7.02	0.04	55.38	6.15	0.14
pO_2_ mmHg	246.00	37.82	181.58	105.81	0.35	135.80	71.00	0.29
sO_2_ (%)	99.70	0.12	97.58	3.10	0.21	90.05	16.60	0.46
Lactate (mmol/L)	2.38	1.69	8.50	3.06	0.01	7.72	4.12	0.24
Troponin (ng/ml)	0.86	0.11				1.10	0.96	0.11

**TABLE 2:** Selected study clinical laboratory values. Chemistry values from Baseline, After REBOA and at End of Study.

	Baseline		After REBOA		End of study	
Chemistry	Mean	SD	Mean	SD	Mean	SD
Na (mmol/L)	140.40	0.89	142.00	2.45	144.80	3.70
K^+^ (mmol/L)	3.90	0.21	3.64	0.81	3.32	0.51
Cl^-^ (mmol/L)	100.60	3.91	107.20	3.27	110.80	6.91
iCa^2+^ (mmol/L)	1.43	0.03	1.59	0.51	1.70	0.51
Glu (mg/dl)	113.60	41.91	121.80	65.50	87.20	45.19
Hb (g/dl)	7.40	1.43	9.00	8.99	7.38	1.79
Anion Gap (mmol/L)	16.60	2.41	18.00	4.93	18.40	4.93

**FIGURE 4:**
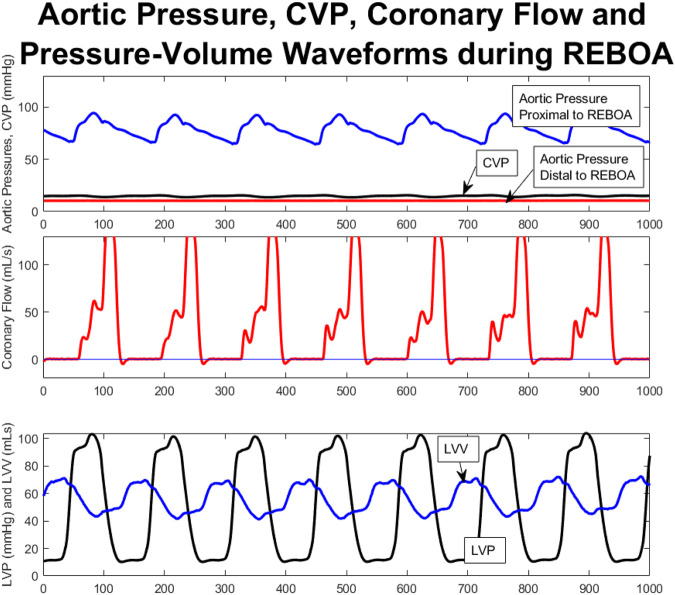
Aortic Pressure, CVP, Coronary Flow and PV Waveforms during total occlusive REBOA catheter placement; representative from a single animal. These are aligned temporarily, with the x axis representing number of 5 ms time steps of the tracing. **(A)**. Aortic pressures monitored proximal to the REBOA (blue), and distal to the REBOA (red) demonstrate complete flattening of the distal aortic pressures to even below the CVP (black) whereas the proximal aortic pressures remain within normal limits and are pulsatile (blue). **(B)**. Coronary flow tracings during occlusive REBOA. Note: Figure 6, Panel three shows the same coronary flow data in an uncropped fashion—juxtaposing this REBOA coronary flow tracing with the coronary flow tracings from the other study period, within this same animal. **(C)**. Decoupled PV loop data showing individual left ventricular pressure and volume tracings over time during occlusive REBOA. *(B) shows the same data as Figure 5C, where here it is aligned against other hemodynamic parameters during REBOA, and in Figure 5C it is aligned against other study periods.

Aortic pressures were monitored proximal and distal to the REBOA catheter balloon. In all five cases when the REBOA was inflated at end of shock the REBOA was occlusive, with flattening of the waveform distal to the REBOA balloon, as shown for a single animal in Figure 4A, where the proximal aortic pressure waveform is appropriately pulsatile and in physiologic range, and the distal aortic pressure waveform has been flattened below the level of the CVP.

### Left Ventricular Function and Contractility

Placement of the occlusive REBOA increased the ESP (59.6 vs. 178.4, *p* = 0.01) but did not affect the ESV, thereby augmenting the ESPVR, Figure 3 and Supplementary Table S1. REBOA also increased arterial elastance (Ea) (1.7 vs. 3, *p* < 0.001), although an associated increase in stroke work was statistically significant prior to adjustment (*p* = 0.024), this effect was not observed after adjustment for multiple comparisons. Graphically, the PV loop shifts down and to the left in pressure-volume space from Baseline to Shock, but with the addition of REBOA the PV loop shifts back toward Baseline. This shift is along the EDPVR curve but is now defined by a steeper ESPVR. This relationship is shown for a single animal in Supplementary Figures S1A,B, In the transition to partial REBOA, this pattern continued. The EDPVR was preserved, see Supplementary Figure S1C, but the ESPVR was shifted again. Here, the ESP (103 mmHg) was lower than during full REBOA (vs. 178.4 mmHg) but remained higher than during Shock (vs. 59.6 mmHg, *p* = 0.002), see Supplementary Table S1.

After transition from Partial REBOA to Recovery, no measures of ESPVR or EDPVR were different than Baseline, with no additional increase in lactate, Tables 1, 2 and Supplementary Figure S2, and normalization of ESP from Shock (59.6 vs. 92.1 mmHg). Qualitatively, PV loops returned to a similar location in the pressure-volume space as seen during Baseline, Supplementary Figure S1C.

**FIGURE 5:**
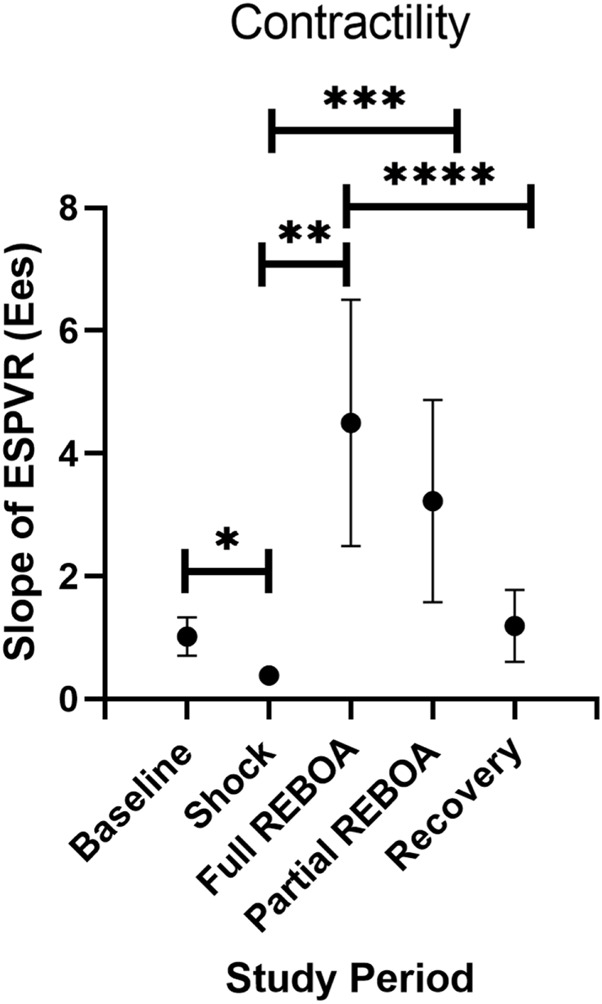
End Systolic Elastance (Ees; the slope of the ESPVR) evolution across study periods. *p = 0.003, **p = 0.002, ***p = 0.01, ****p =0.003.

Mean arterial elastance (Ea: ESP/SV), evolved from study period to study period (One-way Repeated Measures ANOVA, *p* < 0.01), Figure 3B. Mean Ea during Baseline was 2.8 mmHg/ml, compared to 1.7 mmHg/ml during Shock (*p* > 0.05). This increased in the transition from Shock to REBOA (1.7 vs. 3, *p* < 0.001). End Systolic Elastance (Ees; the slope of the ESPVR) evolved throughout this study as well (One-way Repeated Measures ANOVA, *p* = 0.0001), Figure 5. From Baseline to Shock, mean Ees dropped (1.01 vs. 0.39, *p* = 0.003). Accounting for multiple comparisons, placement of REBOA was associated with a substantial increase in Ees compared to Shock (0.39 vs. 4.50, *p* < 0.001). In Partial REBOA, also accounting for multiple comparisons, Ees remained elevated as compared to Shock (3.22 vs. 0.39, *p* = 0.01), and Recovery Ees was lower than fREBOA (1.19 vs. 4.50, *p* = 0.003), but Recovery Ees was not different than Shock (1.19 vs. 0.39, *p* > 0.05).

### Coronary Flow

**FIGURE 6:**
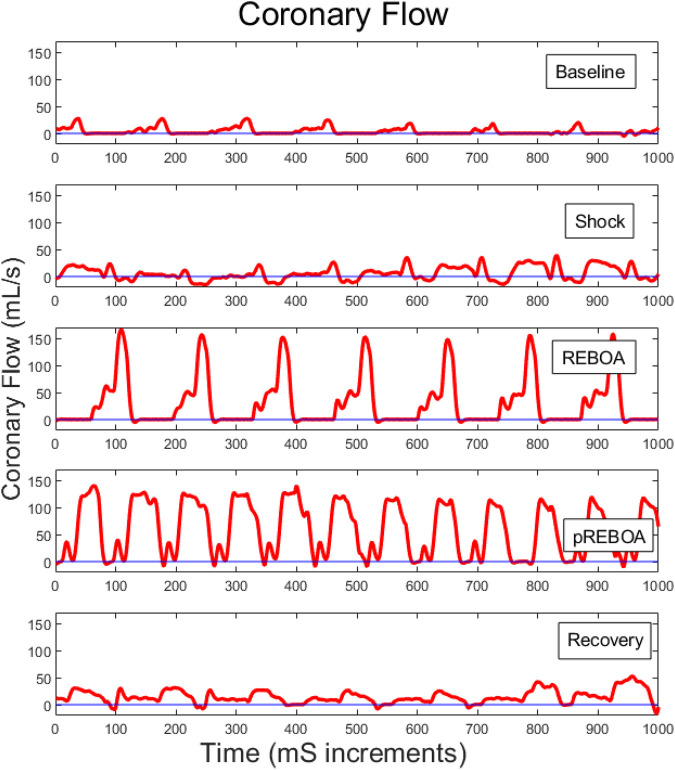
Representative left coronary flow waveforms for one study animal across each of the study periods. Baseline coronary flow wave forms evolve during shock to include a larger percent of each cardiac cycle in retrograde flow. REBOA then rescues this flow reversal but causes substantially higher peak coronary flows. Partial REBOA (pREBOA) has slightly lower peak flows compared to REBOA, but these remain higher than Shock or Baseline, with still little flow reversal. Transition to Recovery period is associated with normalization of coronary flow waveforms.

Coronary flow was measured directly from the left coronary artery throughout the study, and analyzed during each study period for each animal, Figures 4B, 6, and examined alongside pressure and volume tracings from the left ventricular pressure-volume catheter, Figure 4C, and proximal and distal aortic pressure catheters Figure 4A. Examining one animal qualitatively, there was evolution of coronary flow waveforms from study period-to-study period, with obviously higher peaks during REBOA and pREBOA with increased retrograde flow during hemorrhagic shock.

**FIGURE 7:**
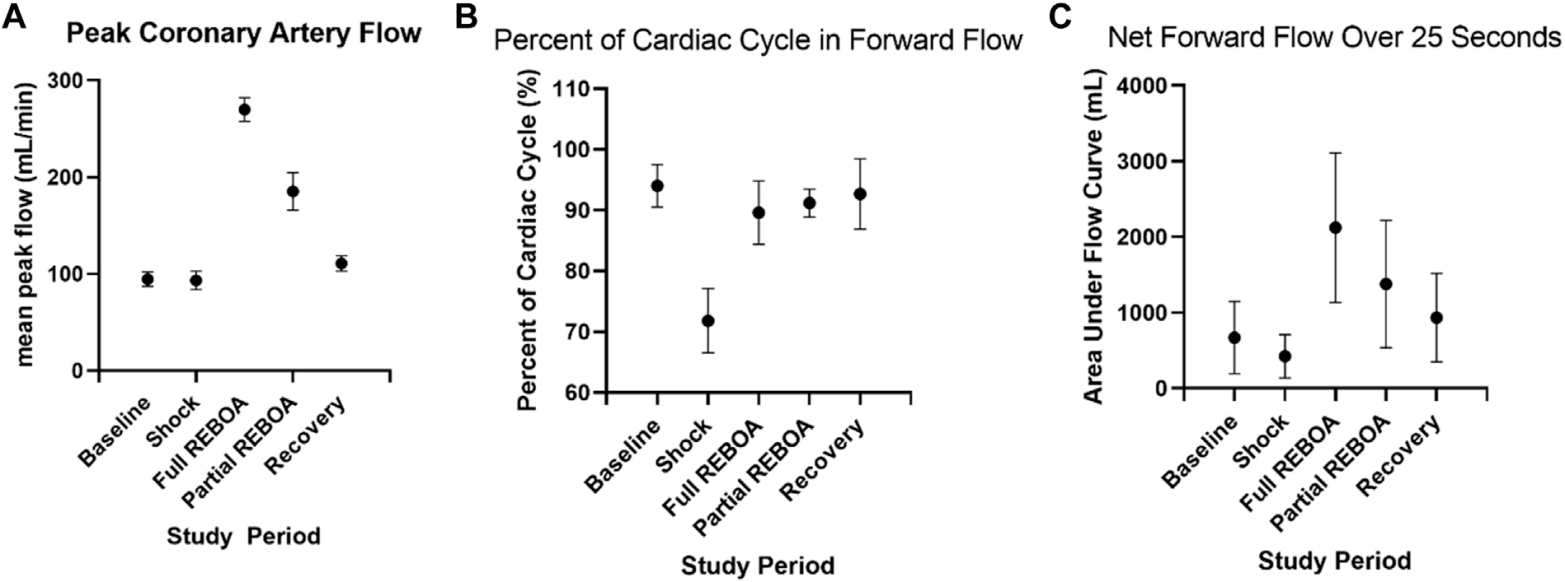
Coronary waveform analysis across study periods. **(A)**. Peak coronary artery flow rate during each study period for all animals. **(B)**. The percent of time spent in antegrade coronary flow during each study period, where flow is reversed more frequently in shock, but is rescued by REBOA. **(C)**. Total net left coronary flow during each study period, over 25 s periods.

A quantitative waveform analysis of peak coronary flow was performed across all animals. Peak coronary flow did not change from Baseline (94.5 ml/min) to Shock (93.4 ml/min), but peak left coronary artery flows were associated with an increase with REBOA (271.2 ml/min, *p* = 0.03 versus shock), with intermediate flows during Partial REBOA (185.3 ml/min), but that did not reach statistical significance (*p* > 0.05 compared to shock), and then normalization of peak flows during Recovery, Figure 7A. Analysis of the percent of time of each cardiac cycle spent in antegrade flow demonstrated more time spent in retrograde flow in Shock as compared to Baseline (94% vs. 71.2%, *p* < 0.001), with restoration of this parameter during REBOA, Partial REBOA and Recovery periods, see Figure 7B. Despite the restoration of the forward flow with REBOA and Partial REBOA, the net forward flow in each cardiac cycle summed over 25 s (5,000 analyzed 5-milisecond timesteps) increased with REBOA compared to Shock (424 ml/5 s period vs. 2126 ml/5 s period, *p* < 0.01), Figure 7C, with intermediate net flow with partial REBOA (1,379 ml/5 s), not reaching statistical significance with full REBOA (*p* = 0.06) or Recovery (*p* = 0.15).

### Histologic Analysis

Pathologic analysis of full thickness left ventricular myocardial samples was examined for signs of injury and ischemia. On one sample the pathologist identified a single cell of ischemic necrosis, Supplementary Figure S3A. The remaining four out of five samples were completely unremarkable, demonstrating no signs of injury or ischemia, Supplementary Figures S3B–E.

## Discussion

REBOA is increasingly used clinically for NCTH following trauma (Morrison et al., 2016; Bulger et al., 2019). As it is use has risen; however, the downsides and complications have become more apparent. In some clinical situations a fully occlusive REBOA is necessary to provide afterload support and hemorrhage control. However, as resuscitation continues, a fully occlusive REBOA may result in proximal hypertension with limited distal perfusion. These data demonstrate that REBOA is associated with supraphysiologic coronary artery flows and LV functional changes. Partial REBOA provided moderate augmentation and has the added benefit (though not evaluated in this study) that it permits some distal perfusion. This has been observed clinically using simple clinical or surrogate measures (e.g., peripheral arterial waveforms or with palpating a distal pulse), but the central cardiovascular function has never been quantified rigorously despite this device’s broadening usage.

In this study, we quantify the LV pump mechanics and coronary flow patterns associated with the transitions from baseline to hemorrhagic shock, then to aortic occlusion with REBOA, then to partially occlusive REBOA, followed by removal of the REBOA entirely and resuscitation. This represents the default clinical progression of REBOA when used in hemorrhagic shock from NCTH. We show that in a model of hemorrhagic shock, transition to partial or full REBOA maintains the EDPVR, but REBOA increases afterload, thereby augmenting the ESPVR and LV contractility. pREBOA provides intermediate augmentation of ESPVR and contractility. REBOA and pREBOA also alter coronary artery blood flow by reversing shock-induced retrograde coronary flow, and increasing peak coronary artery flow, therefore increasing total flow. pREBOA here also shows intermediate augmentation between shock and fREBOA, though this did not reach statistical significance with respect to fREBOA.

Afterload is purported to increase with REBOA (Stannard et al., 2011; Russo et al., 2016; Cannon et al., 2018; Ribeiro Junior et al., 2018; Hoehn et al., 2019; Glaser et al., 2020). However, the extent of this change has never been rigorously quantified, nor has the difference in afterload augmentation between pREBOA and fREBOA been described precisely. Echocardiography has been used to show changes in cardiac function with full REBOA in swine (Teeter et al., 2018), but this is only an estimate within a narrow scope of cardiac biomechanical functional parameters. Other research has demonstrated that Esmolol during REBOA reduces myocardial injury (Hoareau et al., 2020), suggesting that increased afterload is responsible for the cardiovascular effects and injury and that afterload modulation may be an approach to ameliorating the myocardial injury. Other work has captured downstream or surrogate measures of injury but have not demonstrated the underlying mechanism of injury from REBOA (Russo et al., 2016; Sadeghi et al., 2018; Wasicek et al., 2019). Here, we utilize PV loop analysis for the first time to quantify the LV functional changes associated with fREBOA and pREBOA in a hemorrhagic shock model in swine. Though expected, we show that the EDPVR is preserved throughout each of the CRTs; that is, REBOA has little effect on preload. However, the ESPVR curve moves far across the PV plane with each CRT, and the slope of the ESPVR curve (Ees) changes drastically. Here, there is an afterload-recruitable increase in LV contractility secondary to REBOA. The effect of full REBOA here was greater than partial REBOA. This result implies that there is a dose-dependent relationship between the percent of aortic occlusion and change in afterload and, in turn, afterload-dependent LV contractility. Therefore, we show using this gold standard LV functional assessment tool (PV loop) that REBOA is associated with afterload augmentation in hemorrhagic shock and LV contractility. This provides a mechanism for the extant body of work demonstrating downstream myocardial injury from REBOA. Furthermore, this justifies the utility of next generation REBOA catheters with user-independent proximal pressure limits, which formalize pREBOA in a translatable way so that an end-user can more readily apply the physiology demonstrated herein to reduce myocardial injury (Kemp et al., 2021).

To our knowledge, this is the first report also of coronary artery flow measurements during REBOA or during the transition across these CRTs with concomitant measurement of real-time PV loops. We utilized a technical procedure to perform an anterolateral thoracotomy, dissect the left coronary artery off the beating heart to measure flow directly. We found, as expected, increased retrograde flow during shock in the coronary artery, but unexpectedly followed by a substantial increase in peak coronary flow measurements and overall coronary flow beyond physiologic baseline during REBOA, with intermediate flow parameters with pREBOA. Here, peak and total coronary flows were almost three times as high during REBOA than baseline, decreasing then with pREBOA and back to baseline during the recovery period. A similar pattern was seen in Ees. This indicates that the increase in afterload with REBOA results in peak and total coronary artery blood flow proportional to the Ees. This suggests coupling between afterload, coronary blood flow, and afterload-recruitable LV contractility even far outside of the normal physiologic ranges seen at baseline.

In this study with a short follow-up (the study was ended 1 h after first REBOA placement) there was no difference in troponin levels from before and after REBOA placement, nor was there convincing histologic evidence of myocardial ischemia. This is consistent with prior work from our lab which showed troponin peaks at 4 h following 2–4 h of fully occlusive REBOA, with histologic injury also being seen over this longer time period (Wasicek et al., 2019). This prior evaluation caused far more physiologic insult than this work with a prolonged fully occlusive REBOA placement and established that REBOA does cause myocardial injury; in this study the aim was to identify possible mechanisms behind this injury. The findings presented in this article (no troponin increase and essentially no ischemic myocardial injury at 1-h post-REBOA placement) indicate that the surgical dissection of the left coronary artery dissection for flow probe placement caused no injury (i.e., our model and monitoring set-up did not cause any injury, whereas left coronary myocardial territory ischemia and troponin elevation on this timeline could also be caused by coronary injury during instrumentation) and that there was no underlying pathophysiology in these animals that may have confounded these results. Future trials may further evaluate this further by randomizing animals to only partial vs. only full REBOA and follow both for longer, sampling histology from both groups after longer follow-up. This was impossible to investigate here given that all animals underwent both full and partial REBOA.

Overall, these data suggest that a cardioprotective REBOA strategy includes transition from full to partial occlusive as quickly as possible. The supraphysiologic coronary flows and PV loop changes occur shortly after occlusion and being moderate upon transition to partial REBOA. Practically, these data mean, when considering the LV effects and coronary flow, to use a fully occlusive REBOA only when mandatory, with transition to partial REBOA as soon as normotension is restored proximally. For patients who have no perfusing rhythm, or those with uncontrolled hemorrhage, full REBOA should remain the mainstay REBOA strategy, and these were not assessed in this study. For all other patients, consideration should be given to initial partial REBOA, with controlled inflation and monitoring to provide as much afterload support is needed, but not to over-shoot and induce LV effects or supraphysiologic coronary flows. This has the added theoretical benefit of improving also distal perfusion and minimize associated complications, though this was not assessed in this trial.

This study is not without limitations. Of course, it would be unethical and impractical to add coronary flow probes or LV pressure catheters in human patients in severe hemorrhagic shock to measure these LV parameters and investigate coronary flow, whereas swine provide an appropriate cardiovascular model but have some subtle differences compared to humans. Here, we utilized five castrated male swine to approximate a young, relatively healthy cohort as opposed to an elderly cohort that may be more likely to have aortic or cardiac pathology. This strategy was selected to be recapitulate the primary target of interventions on REBOA in trauma: young, healthy males. This strategy, however, neglects other groups on whom REBOA may be applied, such as young women who are subjects of trauma, or from other sources of hemorrhage such as obstetric, iatrogenic or other acute on chronic vascular pathologies. This study may require repetition in a more diverse animal model or to be repeated in other specific cohorts to draw conclusions about the management in other scenarios. The sample size was justified by a power calculation and indeed parameters reached statistical significance, but with this small sample size we saw limited baseline biophysiologic variability. With this sample size the study design was also narrowly focused on these primary outcomes, and so we terminated the study 30 min after the REBOA period, where biologic variability may begin to widen and there would be no clear association between REBOA state and the primary outcomes Future work will evaluate how REBOA and REBOA strategy effect the heart over a longer post-deflation time. We also focused on the most clinically common and therefore clinically relevant progression of REBOA in NCTH: baseline, to shock, to full REBOA, to partial REBOA, to no REBOA again, with start of resuscitation during pREBOA. In other clinical scenarios, the ordering of these may be different, or may go between fREBOA and pREBOA several times, such as if hemorrhage is intermittently poorly controlled, or if blood product administration is variable. This is also important because in our study, with a single ordering, we quickly moved from one experimental phase to the next. In some scenarios 15 min of partial REBOA may or may not be sufficient, such as at a level one trauma center with a readily available operating room versus, for example, a pre-hospital REBOA or one placed in a limited resource setting requiring transfer or increased time to definitive care. This timeframe, however, allowed us to evaluate our outcomes of interest, and a shorter REBOA time allowed for the transfusion of shed blood earlier before temperature changes or other confounders may act. This also allows us to focus the final resuscitation period on the effects of the REBOA and helps limit the confounders that may be introduced from reperfusion injury or ventilatory differences, which were well controlled for through the REBOA period, but may emerge or be harder to control by the end of the study. Anecdotally, we observed the coronary flow and LV PV loop changes in real time, right after the REBOA state was changed. However, it is also possible that some of the observed changes may be related to ischemia-reperfusion injury or possibly related to neuro-hormonal augmentation creating auxiliary mechanisms for the proposed regulatory pathway. Our study design was also not designed to truly test or characterize a dose-response relationship between amount of occlusion by REBOA, and afterload or downstream parameters, though intermediate results with pREBOA here does suggest a stepwise progression to this effect. Lastly, the extent of coronary flow augmentation with REBOA was unexpected. We suggest that this severe extent of coronary flow augmentation across the cardiac capillary bed may cause microvascular injury, providing another mechanism by which afterload may cause cardiac injury other than that of the direct afterload effects demonstrated here more concretely. Overall, the qualitative changes we observed during surgery were drastic and obvious at the bedside, and we chose three parameters in attempt to quantify these qualitative observations: peak coronary flow, the area under the flow curve, and the percent time in retrograde versus antegrade flow. However, these variables are not independent. Future studies may aim to link increased coronary flow more rigorously to myocardial injury and may consider a study design that takes advantage of continuous spectrum of inflation/deflation of the REBOA, with continuous coronary flow monitoring. Here, regression would be an alternate analytic approach, which may further quantify the relationship between the extent of occlusion and coronary flow (e.g., each incremental increase in distal aortic systolic pressure in mmHg may be associated with a relative incremental decrease in peak coronary artery flow, or vice versa). They may also follow these patients post-REBOA for a longer time-period to assess how REBOAs augmentation of basic LV functional parameters leads to longer term myocardial injury, and in turn how they can be ameliorated.

## Conclusion

Overall, REBOA has been thought to convert a large, compliant, aortic reservoir into a smaller, less-compliant space. Prior to this study, however, the association between endovascular aortic occlusion and coronary flow and LV function has not been rigorously demonstrated. This mechanism of REBOA facilitates coronary and head perfusion in severe hypervolemia, but once intravascular volume is restored the LV adjusts to this poorly compliant system by increasing afterload-recruitable contractility. By transitioning to pREBOA, some afterload support is maintained but compliance is now provided once again from the entire vascular reservoir as blood is permitted beyond the balloon. This reduces the deleterious cardiac effects as compared to fREBOA.

We show that partial REBOA sits in a cardiophysiologic middle ground between shock and occlusive REBOA—with intermediate ESPVR, afterload and afterload-recruitable contractility effects, as well as intermediate peak and overall coronary flows. Moreover, we show that many important hemodynamic parameters decreased in shock, are rescued with partial REBOA (e.g., stroke work), suggesting that along with not causing the same myocardial injury as full REBOA, it does provide the same, if not better, resuscitative capability. Partial REBOA facilitates resuscitation and balances this against the cardiovascular injury seen from excessive afterload due to full REBOA. These data suggest that physicians should attempt to wean to partial REBOA as quickly as feasible to prevent undue cardiac injury.

## Data Availability

The raw data supporting the conclusion of this article will be made available by the authors, without undue reservation.

## References

[B1] Abdou H. Elansary N. Poliner D. Patel N. Edwards J. Richmond M. (2021). Development of a Computed Tomography Perfusion Protocol to Support Large Animal Resuscitation Research. J. Trauma Acute Care Surg. 91, 879–885. 10.1097/ta.0000000000003189 33797493

[B2] Bailey Z. S. Cardiff K. Yang X. Gilsdorf J. Shear D. Rasmussen T. E. (2019). The Effects of Balloon Occlusion of the Aorta on Cerebral Blood Flow, Intracranial Pressure, and Brain Tissue Oxygen Tension in a Rodent Model of Penetrating Ballistic-like Brain Injury. Front. Neurol. 10, 1309. 10.3389/fneur.2019.01309 31920932 PMC6930175

[B3] Berland T. L. Veith F. J. Cayne N. S. Mehta M. Mayer D. Lachat M. (2013). Technique of Supraceliac Balloon Control of the Aorta during Endovascular Repair of Ruptured Abdominal Aortic Aneurysms. J. Vasc. Surg. 57, 272–275. 10.1016/j.jvs.2012.09.001 23159478

[B4] Borger van der Burg B. L. S. van Dongen T. T. C. F. Morrison J. J. Hedeman Joosten P. P. A. DuBose J. J. Hörer T. M. (2018). A Systematic Review and Meta-Analysis of the Use of Resuscitative Endovascular Balloon Occlusion of the Aorta in the Management of Major Exsanguination. Eur. J. Trauma Emerg. Surg. 44, 535–550. 10.1007/s00068-018-0959-y 29785654 PMC6096615

[B5] Brenner M. Teeter W. Hoehn M. Pasley J. Hu P. Yang S. (2018). Use of Resuscitative Endovascular Balloon Occlusion of the Aorta for Proximal Aortic Control in Patients with Severe Hemorrhage and Arrest. JAMA Surg. 153, 130–135. 10.1001/jamasurg.2017.3549 28973104 PMC5838921

[B6] Bulger E. M. Perina D. G. Qasim Z. Beldowicz B. Brenner M. Guyette F. (2019). Clinical Use of Resuscitative Endovascular Balloon Occlusion of the Aorta (REBOA) in Civilian Trauma Systems in the USA, 2019: a Joint Statement from the American College of Surgeons Committee on Trauma, the American College of Emergency Physicians, the National Association of Emergency Medical Services Physicians and the National Association of Emergency Medical Technicians. Trauma Surg. Acute Care Open 4, e000376. 10.1136/tsaco-2019-000376 31673635 PMC6802990

[B7] Burlew C. C. Moore E. E. Moore F. A. Coimbra R. McIntyre R. C. Jr Davis J. W. (2012). Western Trauma Association Critical Decisions in Trauma. J. Trauma Acute Care Surg. 73 (6), 1359–1363. 10.1097/TA.0b013e318270d2df 23188227

[B8] Cannon J. Morrison J. Lauer C. Grabo D. Polk T. Blackbourne L. (2018). Resuscitative Endovascular Balloon Occlusion of the Aorta (REBOA) for Hemorrhagic Shock. Mil. Med. 183, 55–59. 10.1093/milmed/usy143 30189087

[B9] Dubose J. J. (2017). How I Do it: Partial Resuscitative Endovascular Balloon Occlusion of the Aorta (P-Reboa). J. Trauma Acute Care Surg. 83, 197–199. 10.1097/ta.0000000000001462 28376018

[B10] Edwards J. Abdou H. Patel N. Madurska M. J. Poe K. Bonin J. E. (2021). The Functional Vascular Anatomy of the Swine for Research. Vascular. 30 (2), 392–402. 10.1177/1708538121996500 33813971

[B11] Edwards J. Elansary N. Stonko D. P. Abdou H. Lang E. Treffalls R. N. (2022). REBOA and its Effect on Hemodynamics and Cerebral Blood Flow as Measured by CTP in a Large Animal Model of Raised Intracranial Pressure and Hemorrhagic Shock. Protoc. Exchange. 10.21203/RS.3.PEX-1653/V1

[B12] Faul F. Erdfelder E. Buchner A. Lang A. G. (2009). Statistical Power Analyses Using G*Power 3.1: Tests for Correlation and Regression Analyses. Behav. Res. Methods 41, 1149–1160. 10.3758/BRM.41.4.1149 19897823

[B13] Glaser J. J. Neidert L. E. Morgan C. G. Brenner M. Stigall K. S. Cardin S. (2020). Resuscitative Endovascular Balloon Occlusion of the Aorta for Thoracic Trauma: A Translational Swine Study. J. Trauma Acute Care Surg. 89, 474–481. 10.1097/ta.0000000000002749 32345903

[B14] Heindl S. E. Wiltshire D. A. Vahora I. S. Tsouklidis N. Khan S. (2020). Partial versus Complete Resuscitative Endovascular Balloon Occlusion of the Aorta in Exsanguinating Trauma Patients with Non-compressible Torso Hemorrhage. Cureus 12, e8999. 10.7759/cureus.8999 32775079 PMC7402546

[B15] Hoareau G. L. Beyer C. A. Caples C. M. Spruce M. W. Gilbert Z. Grayson J. K. (2020). Esmolol Reduces Myocardial Injury Induced by Resuscitative Endovascular Balloon Occlusion of the Aorta (REBOA) in a Porcine Model of Hemorrhagic Shock. Injury 51, 2165–2171. 10.1016/j.injury.2020.07.005 32669205

[B16] Hoehn M. R. Teeter W. A. Morrison J. J. Gamble W. B. Hu P. Stein D. M. (2019). Aortic branch Vessel Flow during Resuscitative Endovascular Balloon Occlusion of the Aorta. J. Trauma Acute Care Surg. 86, 79–85. 10.1097/ta.0000000000002075 30252777

[B17] Hughes C. W. (1954). Use of an Intra-aortic Balloon Catheter Tamponade for Controlling Intra-abdominal Hemorrhage in Man. Surgery 36, 65–68. 10.5555/URI:PII:0039606054902664 13178946

[B18] Hughes M. Perkins Z. (2020). Outcomes Following Resuscitative Thoracotomy for Abdominal Exsanguination, a Systematic Review. Scand. J. Trauma Resusc Emerg. Med. 28 (1 28), 9–10. 10.1186/s13049-020-0705-4 32028977 PMC7006065

[B19] Inaba K. Chouliaras K. Zakaluzny S. Swadron S. Mailhot T. Seif D. (2015). FAST Ultrasound Examination as a Predictor of Outcomes after Resuscitative Thoracotomy: A Prospective Evaluation. Ann. Surg. 262, 512–518. 10.1097/sla.0000000000001421 26258320

[B20] Ivatury R. R. Kazigo J. Rohman M. Gaudino J. Simon R. Stahl W. M. (1991). "Directed" Emergency Room Thoracotomy: a Prognostic Prerequisite for Survival. J. Trauma Inj. Infect. Crit. Care. 31, 1076–1082. 10.1097/00005373-199108000-00005 1875433

[B21] Johnson M. A. Williams T. K. Ferencz S.-A. E. Davidson A. J. Russo R. M. O’Brien W. T. Sr (2017). The Effect of Resuscitative Endovascular Balloon Occlusion of the Aorta, Partial Aortic Occlusion and Aggressive Blood Transfusion on Traumatic Brain Injury in a Swine Multiple Injuries Model. J. Trauma acute Care Surg. 83, 61–70. 10.1097/ta.0000000000001518 28632582 PMC5505178

[B22] Johnson M. A. Neff L. P. Williams T. K. DuBose J. J. EVAC Study Group (2016). Partial Resuscitative Balloon Occlusion of the Aorta (P-REBOA): Clinical Technique and Rationale. J. Trauma Acute Care Surg. 81, S133–S137. 10.1097/ta.0000000000001146 27244578 PMC8789541

[B23] Kemp M. T. Wakam G. K. Williams A. M. Biesterveld B. E. O’Connell R. L. Vercruysse C. A. (2021). A Novel Partial Resuscitative Endovascular Balloon Aortic Occlusion Device that Can Be Deployed in Zone 1 for More Than 2 hours with Minimal Provider Titration. J. Trauma Acute Care Surg. 90, 426–433. 10.1097/ta.0000000000003042 33492106

[B24] Kisat M. Morrison J. J. Hashmi Z. G. Efron D. T. Rasmussen T. E. Haider A. H. (2013). Epidemiology and Outcomes of Non-compressible Torso Hemorrhage. J. Surg. Res. 184, 414–421. 10.1016/j.jss.2013.05.099 23831230

[B26] Madurska M. J. Abdou H. Richmond M. J. Elansary N. N. Wong P. F. Rasmussen T. E. (2020). Development of a Selective Aortic Arch Perfusion System in a Porcine Model of Exsanguination Cardiac Arrest. J. Vis. Exp. 25, e61573. 10.3791/61573 32925879

[B27] Madurska M. J. Ross J. D. Scalea T. M. Morrison J. J. (2021a). State-of-the-Art Review-Endovascular Resuscitation. Shock (Augusta, Ga.) 55, 288–300. 10.1097/shk.0000000000001636 32925603

[B28] Madurska M. J. Abdou H. Leung L. Y. Richmond M. J. Elansary N. N. Scalea T. M. (2021b). The Cardiac Physiology Underpinning Exsanguination Cardiac Arrest: Targets for Endovascular Resuscitation. Shock. 55, 83–89. 10.1097/shk.0000000000001607 33337788

[B29] Malina M. Veith F. Ivancev K. Sonesson B. (2005). Balloon Occlusion of the Aorta during Endovascular Repair of Ruptured Abdominal Aortic Aneurysm. J. Endovascular Ther. 12, 556–559. 10.1583/05-1587.1 16212455

[B30] Mazzorana V. Smith R. S. Morabito D. J. Brar H. S. (1994). Limited Utility of Emergency Department Thoracotomy. Am. Surg. 60, 516–521. 8010566

[B31] Morrison J. J. Stannard A. Rasmussen T. E. Jansen J. O. Tai N. R. Midwinter M. J. (2013a). Injury Pattern and Mortality of Noncompressible Torso Hemorrhage in UK Combat Casualties. J. Trauma Acute Care Surg. 75, S263–S268. 10.1097/TA.0b013e318299da0a 23883918

[B32] Morrison J. J. Poon H. Rasmussen T. E. Khan M. A. Midwinter M. J. Blackbourne L. H. (2013b). Resuscitative Thoracotomy Following Wartime Injury. J. Trauma Acute Care Surg. 74, 825–829. 10.1097/ta.0b013e31827e1d26 23425742

[B33] Morrison J. J. Galgon R. E. Jansen J. O. Cannon J. W. Rasmussen T. E. Eliason J. L. (2016). A Systematic Review of the Use of Resuscitative Endovascular Balloon Occlusion of the Aorta in the Management of Hemorrhagic Shock. J. Trauma acute Care Surg. 80, 324–334. 10.1097/ta.0000000000000913 26816219

[B34] Morrison J. J. (2017). Noncompressible Torso Hemorrhage. Crit. Care Clin. 33, 37–54. 10.1016/j.ccc.2016.09.001 27894498

[B35] Morrison J. J. Percival T. J. Markov N. P. Villamaria C. Scott D. J. Saches K. A. (2012). Aortic Balloon Occlusion Is Effective in Controlling Pelvic Hemorrhage. J. Surg. Res. 177, 341–347. 10.1016/j.jss.2012.04.035 22591921

[B36] Morrison J. J. Rasmussen T. E. (2012). Noncompressible Torso Hemorrhage. Surg. Clin. North America 92, 843–858. 10.1016/j.suc.2012.05.002 22850150

[B37] Morrison J. J. Ross J. D. Houston R. Watson J. D. B. Sokol K. K. Rasmussen T. E. (2014). Use of Resuscitative Endovascular Balloon Occlusion of the Aorta in a Highly Lethal Model of Noncompressible Torso Hemorrhage. Shock 41, 130–137. 10.1097/shk.0000000000000085 24430492

[B38] Nowadly C. D. Johnson M. A. Hoareau G. L. Manning J. E. Daley J. I. (2020). The Use of Resuscitative Endovascular Balloon Occlusion of the Aorta (REBOA) for Non‐traumatic Cardiac Arrest: A Review. J. Am. Coll. Emerg. Physicians Open. 1, 737–743. 10.1002/emp2.12241 33145513 PMC7593442

[B39] Olsen M. H. Thonghong T. Søndergaard L. Møller K. (2020). Standardized Distances for Placement of REBOA in Patients with Aortic Stenosis. Sci. Rep. 10 (1 10), 13410–13417. 10.1038/s41598-020-70364-9 32770039 PMC7414869

[B40] Patel N. Abdou H. Edwards J. Elansary N. N. Poe K. Richmond M. J. (2021). Measuring Cardiac Output in a Swine Model. J. Vis. Exp. (171), e62333. 10.3791/62333 34057452

[B41] Ribeiro Junior M. A. F. Feng C. Y. D. Nguyen A. T. M. Rodrigues V. C. Bechara G. E. K. de-Moura R. R. (2018). The Complications Associated with Resuscitative Endovascular Balloon Occlusion of the Aorta (REBOA). World J. Emerg. Surg. 13, 20–26. 10.1186/s13017-018-0181-6 29774048 PMC5948672

[B42] Russo R. M. White J. M. Baer D. G. (2021). Partial Resuscitative Endovascular Balloon Occlusion of the Aorta: A Systematic Review of the Preclinical and Clinical Literature. J. Surg. Res. 262, 101–114. 10.1016/j.jss.2020.12.054 33561721

[B43] Russo R. M. Neff L. P. Lamb C. M. Cannon J. W. Galante J. M. Clement N. F. (2016). Partial Resuscitative Endovascular Balloon Occlusion of the Aorta in Swine Model of Hemorrhagic Shock. J. Am. Coll. Surgeons 223, 359–368. 10.1016/j.jamcollsurg.2016.04.037 27138649

[B44] Sadeghi M. Hörer T. M. Forsman D. Dogan E. M. Jansson K. Kindler C. (2018). Blood Pressure Targeting by Partial REBOA Is Possible in Severe Hemorrhagic Shock in Pigs and Produces Less Circulatory, Metabolic and Inflammatory Sequelae Than Total REBOA. Injury 49, 2132–2141. 10.1016/j.injury.2018.09.052 30301556

[B45] Stannard A. Eliason J. L. Rasmussen T. E. (2011). Resuscitative Endovascular Balloon Occlusion of the Aorta (REBOA) as an Adjunct for Hemorrhagic Shock. J. Trauma - Inj. Infect. Crit. Care 71, 1869–1872. 10.1097/ta.0b013e31823fe90c 22182896

[B46] Stannard A. Morrison J. J. Scott D. J. Ivatury R. A. Ross J. D. Rasmussen T. E. (2013). The Epidemiology of Noncompressible Torso Hemorrhage in the Wars in Iraq and Afghanistan. J. Trauma Acute Care Surg. 74 (3), 830–834. 10.1097/TA.0b013e31827a3704 23425743

[B47] Stokes S. C. Theodorou C. M. Zakaluzny S. A. DuBose J. J. Russo R. M. (2021). Resuscitative Endovascular Balloon Occlusion of the Aorta in Combat Casualties: The Past, Present, and Future. J. Trauma Acute Care Surg. 91, S56–S64. 10.1097/ta.0000000000003166 33797487 PMC8324517

[B49] Stonko D. P. Edwards J. Abdou H. Elansary N. N. Lang E. Savidge S. G. (2022). A Technical and Data Analytic Approach to Pressure-Volume Loops over Numerous Cardiac Cycles. JVS: Vasc. Sci. 3, 73–84. 10.1016/J.JVSSCI.2021.12.003 35257117 PMC8897635

[B50] Stonko D. P. Edwards J. Abdou H. Elansary N. N. Lang E. Savidge S. G. (2021). PV Loops and REBOA during Hemorrhage and Resuscitation. Protoc. Exchange. 10.21203/RS.3.PEX-1646/V1

[B51] Teeter W. A. Conti B. M. Wasicek P. J. Morrison J. J. Parsell D. Gamble B. (2018). Feasibility of Basic Transesophageal Echocardiography in Hemorrhagic Shock: Potential Applications during Resuscitative Endovascular Balloon Occlusion of the Aorta (REBOA). Cardiovasc. Ultrasound 16 (1), 12. 10.1186/s12947-018-0129-8 30012168 PMC6048745

[B52] Uchino H. Tamura N. Echigoya R. Ikegami T. Fukuoka T. (2016). “Reboa” – is it Really Safe? A Case with Massive Intracranial Hemorrhage Possibly due to Endovascular Balloon Occlusion of the Aorta (Reboa). Am. J. Case Rep. 17, 810–813. 10.12659/ajcr.900267 27799653 PMC5091201

[B53] Wasicek P. J. Teeter W. A. Yang S. Banchs H. Galvagno S. M. Hu P. (2019). Extended Resuscitative Endovascular Balloon Occlusion of the Aorta (REBOA)-induced Type 2 Myocardial Ischemia: a Time-Dependent Penalty. Trauma Surg. Acute Care Open 4, e000194. 10.1136/tsaco-2018-000194 30815536 PMC6361364

[B54] White J. M. Cannon J. W. Stannard A. Markov N. P. Spencer J. R. Rasmussen T. E. (2011). Endovascular Balloon Occlusion of the Aorta Is superior to Resuscitative Thoracotomy with Aortic Clamping in a Porcine Model of Hemorrhagic Shock. Surgery. 150, 400–409. 10.1016/j.surg.2011.06.010 21878225

