# Supplementary material for: The Underlying Cardiovascular Mechanisms of Resuscitation and Injury of REBOA and Partial REBOA
Source: Front Physiol. 2022 May 9;13:871073. doi: 10.3389/fphys.2022.871073 (PMC9125334; doi:10.3389/fphys.2022.871073)

**Supplemental Figure 1.** Left Ventricular Pressure-Volume loop evolution over the study period. A. Baseline Pressure-Volume loop (blue, right) with average baseline pressure-volume loop (red, right). The animal then hemorrhaged to SPB 45 mmHg. Pressure-volume loop in hemorrhagic shock (blue, left) and average PV loop while in hemorrhagic shock (left, red). B. Includes the same Baseline and Shock PV loops, but includes the next study period PV loop, with Full REBOA (middle blue), with average full REBOA loop (red, middle). C. The average PV loops for all 5 study periods, including the first 3 seen in panel B but also partial REBOA and the recovery period. The EDPVR is clearly preserved across study period, but the ESPVR is not.

**
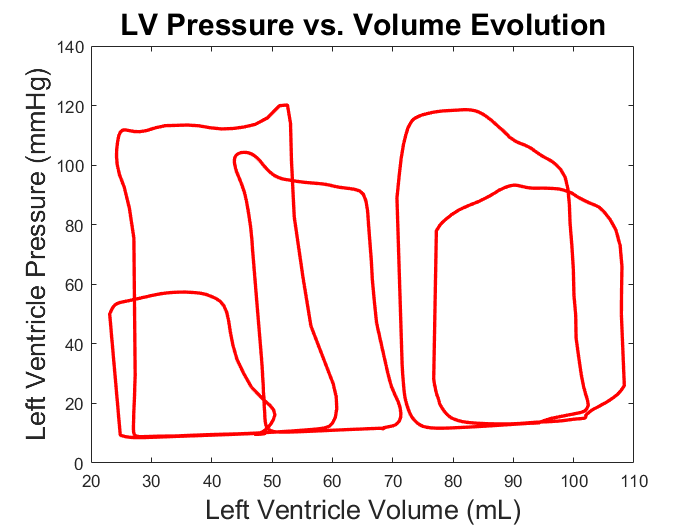

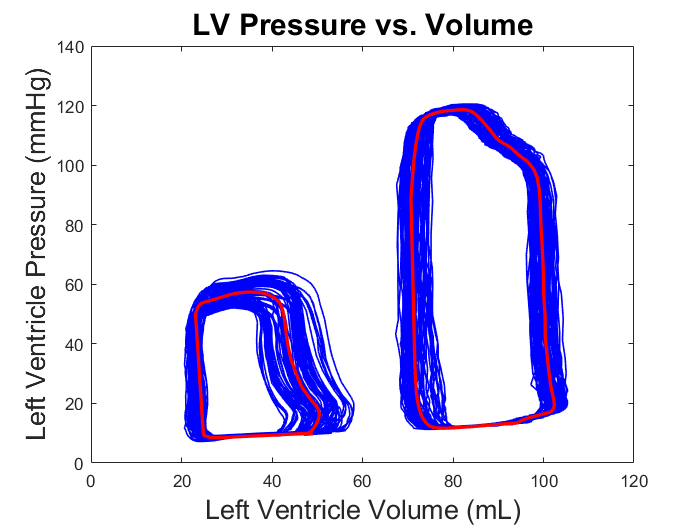
**

After

Recovery

Baseline

Hem.

Shock

Full REBOA

Partial REBOA

Hemorrhagic

Shock

Baseline

Baseline

Hem.

Shock

Full REBOA

**Supplemental Figure 2. Lactic acid trends during the study.** From baseline to post-REBOA lactate peaked from median 1.5 mmol/L to median 6.9 mmol/L. Lactic acid did not continue to increase throughout the recovery period and ended without further uptrend.


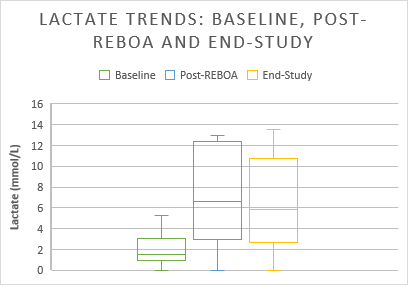


P=0.01

**Supplemental Table 1. A.** Hemodynamic parameters (Mean, SD) taken from each study period. **B.** p-values comparing study period to study period across selected important HD parameters.

|  | **Baseline** |  | **Shock** |  | **Full REBOA** | | **Partial REBOA** | | **Recovery** |  |
| --- | --- | --- | --- | --- | --- | --- | --- | --- | --- | --- |
|  | Mean | SD | Mean | SD | Mean | SD | Mean | SD | Mean | SD |
| **HR** | 94.3 | 10.9 | 99.1 | 3.2 | 111.8 | 25.5 | 123.1 | 20.1 | 90.6 | 7.5 |
| **SV** | 39.9 | 5.2 | 37.4 | 11.7 | 62.1 | 21.6 | 52.6 | 24.2 | 64.0 | 39.3 |
| **SW** | 3101.8 | 688.9 | 1174.6 | 264.5 | 4318.0 | 2730.9 | 4195.2 | 2413.5 | 4259.0 | 2651.4 |
| **EDV** | 95.8 | 8.5 | 73.5 | 28.1 | 95.5 | 26.9 | 90.9 | 24.3 | 145.2 | 58.7 |
| **ESV** | 61.3 | 11.5 | 39.4 | 22.2 | 42.2 | 11.2 | 40.9 | 24.6 | 87.7 | 30.0 |
| **EDP** | 14.8 | 3.4 | 12.7 | 3.1 | 14.7 | 3.2 | 15.6 | 4.0 | 17.8 | 4.5 |
| **ESP** | 109.8 | 27.5 | 59.6 | 5.5 | 178.4 | 65.7 | 103.0 | 26.8 | 92.1 | 12.6 |
| **EF** | 42.1 | 6.2 | 53.2 | 16.0 | 64.7 | 17.2 | 58.8 | 21.3 | 41.9 | 14.7 |
| **CO** | 3771.8 | 674.5 | 3708.0 | 1168.9 | 7136.8 | 3224.3 | 6673.0 | 3556.5 | 5929.0 | 3941.5 |
| **Ea** | 2.8 | 0.8 | 1.7 | 0.5 | 3.0 | 0.7 | 2.2 | 0.8 | 1.9 | 1.0 |

|  |  |  |  |  |  |
| --- | --- | --- | --- | --- | --- |
| **HR** |  | Shock | Full REBOA | Partial REBOA | Recovery |
| Baseline |  | 0.358616 | 0.271853 | 0.10336 | 0.533081 |
| Shock |  |  | 0.29456 | 0.070252 | 0.075273 |
| Full REBOA | |  |  | 0.471939 | 0.106397 |
| Partial REBOA | |  |  |  | 0.037462 |
|  |  |  |  |  |  |
| **SW** |  | Shock | Full REBOA | Partial REBOA | Recovery |
| Baseline |  | 0.006439 | 0.395354 | 0.269957 | 0.291921 |
| Shock |  |  | 0.047229 | 0.154696 | 0.06641 |
| Full REBOA | |  |  | 0.941776 | 0.97628 |
| Partial REBOA | |  |  |  | 0.941042 |
|  |  |  |  |  |  |
| **EA** |  | Shock | Full REBOA | Partial REBOA | Recovery |
| Baseline |  | 0.084142 | 0.720667 | 0.225118 | 0.19254 |
| Shock |  |  | 0.001221 | 0.323945 | 0.735239 |
| Full REBOA | |  |  | 0.126185 | 0.094445 |
| Partial REBOA | |  |  |  | 0.160535 |
|  |  |  |  |  |  |
| **ESV** |  | Shock | Full REBOA | Partial REBOA | Recovery |
| Baseline |  | 0.077554 | 0.017509 | 0.134959 | 0.080062 |
| Shock |  |  | 0.687566 | 0.895567 | 0.001082 |
| Full REBOA | |  |  | 0.919342 | 0.006477 |
| Partial REBOA | |  |  |  | 0.040765 |
|  |  |  |  |  |  |
| **ESP** |  | Shock | Full REBOA | Partial REBOA | Recovery |
| Baseline |  | 0.024817 | 0.099312 | 0.25614 | 0.246381 |
| Shock |  |  | 0.013643 | 0.035159 | 0.004823 |
| Full REBOA | |  |  | 0.063076 | 0.036115 |
| Partial REBOA | |  |  |  | 0.363731 |

**Supplemental Figure 3.** Representative full thickness end-of-study myocardial biopsies, one from each of the five animals (A-E). Of all cells on sampled slides across the entire 5 samples, only one cell of necrosis was identified (Supp Figure 3A)

**
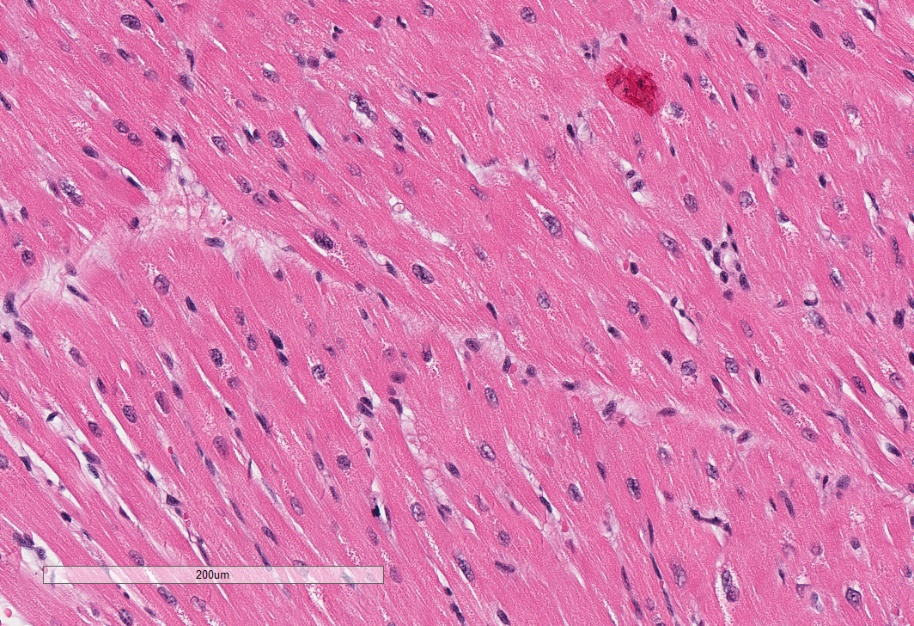
**A.

B.


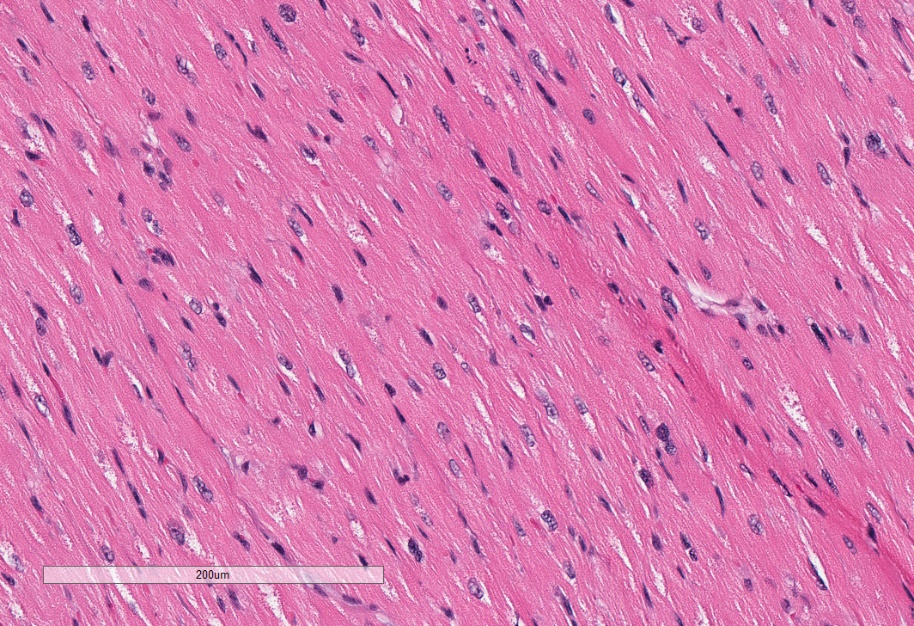


**C.**


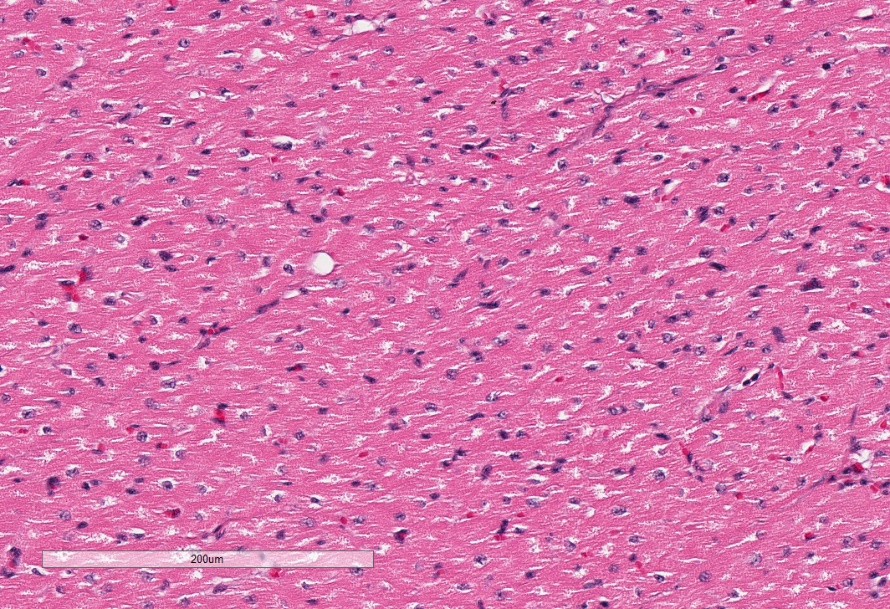


D.


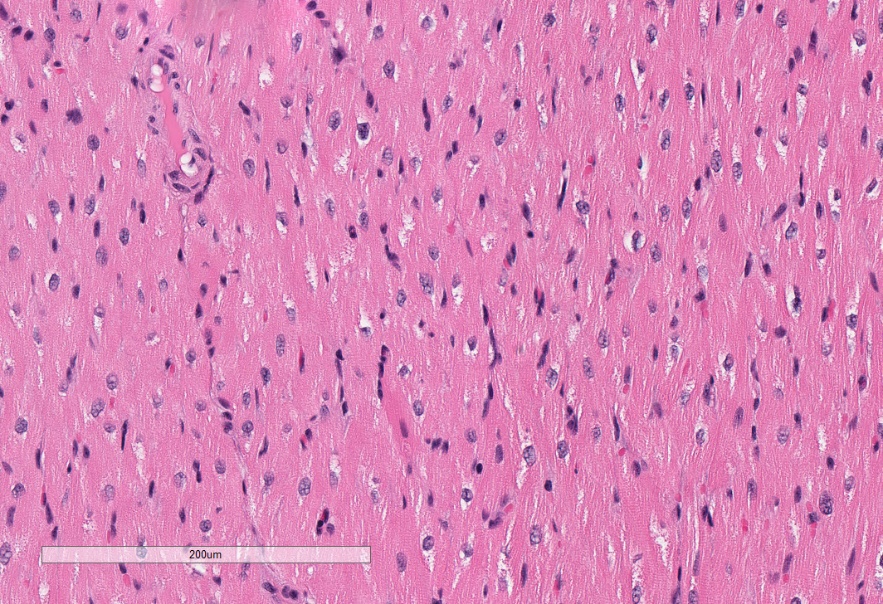


**E.**


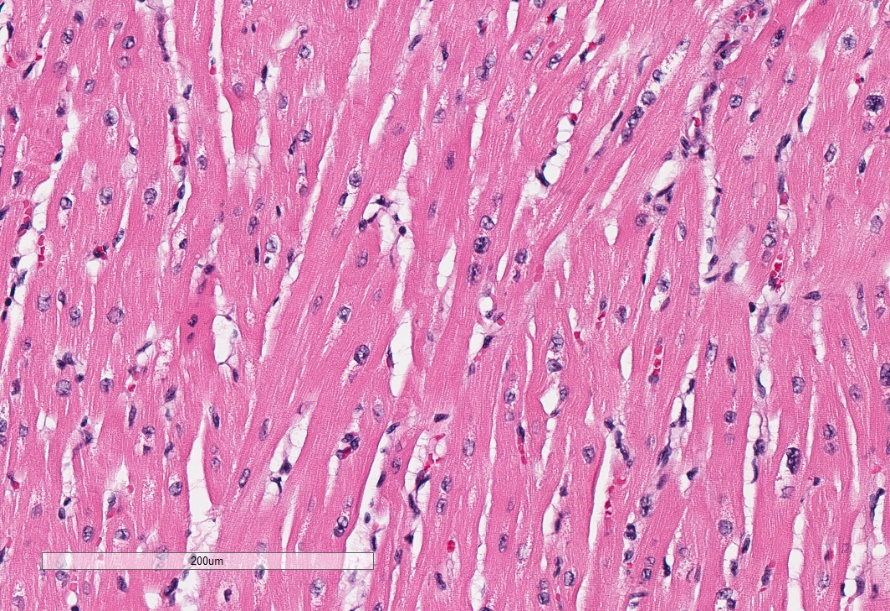

Supplement: Supplementary file 1 [file DataSheet1.docx]
